# The long-term outcomes following the use of inactivated autograft in the treatment of primary malignant musculoskeletal tumor

**DOI:** 10.1186/s13018-015-0324-3

**Published:** 2015-11-17

**Authors:** Jielai Yang, Bin Zhu, Kai Fu, Qingcheng Yang

**Affiliations:** Department of Orthopedics, Shanghai Jiao Tong University Affiliated Sixth People’s Hospital, Shanghai, 200233 China

**Keywords:** Malignant bone tumor, Limb salvage, Recycling autograft, Alcohol

## Abstract

**Background:**

Biological reconstruction surgery is a tough but alluring option for treating primary malignant musculoskeletal tumors. In this article, we evaluate the clinical outcomes of primary malignant musculoskeletal tumors treated with inactivated autograft using alcohol.

**Method:**

In this article, we include 58 patients who had primary malignant bone tumors treated with wide resection and recycling autograft reconstruction using alcohol between January 2003 and January 2013. The outcomes were measured by recurrence, functional status, and complications. Functional status was assessed according to the Musculoskeletal Tumor Society Score (MSTSS). The Kaplan-Meier survival curve was used to evaluate the survival rate of the patient.

**Result:**

The most common tumor was osteosarcoma (31 cases) followed by chondrosarcoma (10 cases). The tibia was the most frequently involved skeletal site (27 cases) followed by femur (26 cases). The median follow-up period was 54 months, ranging from 18 to 96 months. In 58 patients, 12 were with local recurrence (20.7 %), 16 with lung metastasis (27.6 %), and 13 with complications (22.4 %). The main complication was infection (8 cases). The autografts survived in 49 patients (84.5 %). The mean MSTSS score was 78.5 %, ranging from 47 to 98 %.

**Conclusion:**

Recycling autograft reconstruction using alcohol had favorable clinical outcomes to some degree; however, the recurrence and complication rates seem to be high. Thus, we should apply this method with caution and choose the patients with strict surgical indication.

## Background

Malignant bone tumors have characteristics of high degree of malignancy and high rates of recurrence, morbidity, and early metastasis. Since the 1970s, with the advances in diagnostic imaging, chemo- and radiotherapy, and operative techniques, limb salvage surgery has become a preferred choice for malignant bone tumor [1].

Wide resection and limb salvage surgery are considered a standard treatment for primary malignant musculoskeletal tumors [2–4]. Compared with amputation, limb salvage surgery did not reduce survival rate. In contrast, it achieved lower rate of local recurrence and retained part of limb function [5]. The options for reconstruction following tumor excision include endoprostheses [6], allografting [7], composite arthroplasty [8], and distraction osteogenesis [9]. Tumor prosthesis reconstruction gives the most favorable clinical result in terms of functional outcome and complication rates. However, endoprosthesis has the limitation of long-term survival of prosthesis and high cost. Unfortunately, most patients in developing countries cannot afford this type of reconstruction and are treated with amputation. Biological limb salvage procedures are considered an alternative treatment for patients who cannot afford endoprosthesis. Recycling of the resected segment is one type of biological reconstruction. Several methods have been applied including autoclaving [10], freezing [11], pasteurization [12], extracorporeal irradiation [13], and alcohol inactivation [14, 15]. In China, we primarily choose inactivated autograft using alcohol to carry out biological limb salvage procedures. The method has several advantages, with material easily obtained, economic, no rejection, and low infection rate.

However, there still remain some serious problems. The aim of this study was to evaluate the long-term outcome following the use of this method and then put forward some constructive advice to optimize it.

## Patients and methods

This was a single-centered and retrospective study approved by the Ethics Committee of Shanghai Jiao Tong University Affiliated Sixth People’s Hospital. All procedures were in compliance with the Helsinki Declaration. Informed consent for participation was obtained from all participants in this study. We reviewed 58 patients who had a primary malignant bone tumor treated with wide resection and autograft reconstruction using alcohol. The operation was performed between January 2003 and January 2013 (Table 1).

**Table 1:** Details of patients who underwent reconstruction with inactivated autograft treated with alcohol

Case	Gender and age(Y)	Location	Stage^a^	Histology	Length, cm	Resection	MSTSS score ,%	Outcomes	Follow-up (months)
1	M/12	DF	IIB	OSA	15	IA	81	Death	29
2	M/16	PT	IIB	OSA	18	IA	92	DF	61
3	F/21	PT	IIB	CHOS	21	IA	95	DF	70
4	M/12	DF	IIB	OSA	17	IA	81	DF	78
5	F/13	PT	IIB	OSA	23	IC	73	DF	69
6	F/17	PT	IIB	OSA	16	IA	85	DF	73
7	M/2O	PF	IIB	SSA	25	IA	69	Death	23
8	M/16	DF	IB	OSA	18	IC	67	DF	90
9	M/32	PT	IIB	CHOS	16	IA	98	DF	84
10	F/11	PH	IB	OSA	27	IA	87	DF	96
11	F/15	SH	IIB	0SA	21	IC	89	Death	40
12	M/19	DF	IIB	SSA	15	EA	72	Death	43
13	M/14	DF	IIB	EWS	18	IC	78	Death	48
14	M/23	PT	IIB	CHOS	19	IA	91	DF	72
15	M/17	PF	IIB	OSA	31	IA	76	DF	68
16	F/13	DF	IIB	OSA	22	EA	80	Death	55
17	F/19	PT	IB	IGTB	19	IC	77	DF	88
18	M/39	PT	IB	CHOS	17	IA	96	Death	56
19	M/26	DF	IIB	CHOS	22	IA	72	DF	60
20	M/21	PT	IIB	MCHOS	23	IA	94	DF	62
21	F/15	DF	IIB	OSA	28	IA	85	Death	36
22	F/14	PF	IB	IGTB	24	IA	70	Death	29
23	M/15	PT	IIB	OSA	16	IC	87	DF	70
24	M/26	DF	IIB	MCHOS	17	IA	85	Death	18
25	F/18	PT	IIB	OSA	19	IC	78	DF	79
26	M/2O	DF	IIB	FSA	18	IA	67	Death	28
27	F/13	DF	IIB	EWS	30	IA	54	Death	41
28	F/16	PT	IB	OSA	21	IA	86	Death	38
29	M/25	PT	IIB	CHOS	25	IA	74	DF	64
30	M/17	PH	IIB	OSA	17	EA	47	Death	28
31	F/12	DF	IB	OSA	19	IA	86	DF	65
32	M/15	PT	IIB	IGTB	23	IA	95	DF	96
33	M/19	DF	IIB	FSA	29	IA	82	Death	29
34	F/22	PT	IIB	MCHOS	22	IA	83	Death	38
35	F/13	DF	IB	OSA	25	IC	70	DF	80
36	M/18	PT	IIB	OSA	18	EA	93	Death	34
37	F/45	DF	IIB	CHOS	15	IA	85	DF	70
38	F/16	PT	IIB	0SA	19	IA	67	DF	66
39	M/19	DF	IIB	SSA	21	IA	94	Death	53
40	M/13	PT	IIB	MFCT	29	IA	73	Death	48
41	M/21	DF	IB	CHOS	18	IC	49	Death	36
42	M/14	PH	IIB	OSA	16	IA	85	Death	52
43	M/19	DF	IIB	OSA	14	IA	79	DF	78
44	M/2O	PT	IB	MCHOS	19	IA	70	Death	30
45	F/12	DF	IIB	OSA	21	IA	81	Death	42
46	F/17	PT	IIB	OSA	29	IA	58	Death	23
47	M/15	PT	IIB	OSA	31	IA	84	DF	88
48	M/21	DF	IB	MCHOS	17	IA	78	Death	55
49	F/16	PH	IIB	OSA	19	IC	59	Death	28
50	F/19	PT	IIB	SSA	21	IA	80	Death	34
51	F/11	DF	IIB	OSA	27	IA	71	Death	33
52	F/17	PT	IIB	OSA	22	IA	97	DF	82
53	M/38	PT	IB	CHOS	25	IA	71	Death	46
54	M/12	DF	IIB	OSA	17	IC	93	Death	58
55	M/15	PT	IIB	MFCT	19	IA	48	Death	19
56	F/11	DF	IIB	OSA	26	IA	75	DF	72
57	F/24	PT	IB	CHOS	19	IC	79	Death	46
58	M/13	PT	IIB	SSA	17	IA	83	Death	55

*DF* distal femur, *PF* proximal femur, *PH* proximal humerus, *PT* proximal tibia, *SH* shaft humerus, *OSA* osteosarcoma, *CHOS* chondrosarcoma, *MCHOS* mucus chondrosarcoma, *FSA* fibrosarcoma, *EWS* Ewing’s sarcoma, *IGTB* invasive giant cell tumor of bone, *MFCT* malignant fibrous cell tumors, *SSA* synovial sarcoma, *EA* extra-articular, *IA* intra-articular, *IC* intercalary, *DF* disease free

^a^Enneking surgical stage

The mean follow-up period for the patients was 54.3 months (18–96). Of the 58 cases (33 men), the most common tumor was osteosarcoma (31 cases) followed by chondrosarcoma (10 cases); the tibia was the most frequently involved skeletal site (27 cases) followed by the femur (26 cases). All had a histological diagnosis based on incisional biopsy (Table 2).

**Table 2:** Histological diagnosis of inactivated autografts used as reconstruction after the excision of a tumor

Histological diagnosis	No. of patients (*n* = 58)
Osteosarcoma OSA	31
Chondrosarcoma CHOS	10
Mucus chondrosarcoma MCHOS	5
Fibrosarcoma FSA	2
Ewing’s sarcoma EWS	2
Invasive giant cell tumor of bone IGTB	2
Malignant fibrous cell tumors MFCT	2
Synovial sarcoma SSA	4

The primary tumor was evaluated on plain radiographs, computed tomography (CT) scans, and magnetic resonance imaging scans. The bone scintigraphy and CT scanning of the chest were performed to confirm that there were no metastases. All the patients received the 2–3 circles of standard three-course neoadjuvant chemotherapy with a 3-week interval between cycles. After receiving the full course of neoadjuvant chemotherapy, all the patients were restaged using MRI and received surgery 2 weeks after the last course. Postoperative chemotherapy (1 circle) was performed every month and lasted for 12 to 18 months.

### Operative technique

Wide resection was performed on all patients. The level of resection was determined by the preoperative MRI (restaging MRI) and an intraoperative fluoroscopic image. The surgical technique was described as follows: (1) the lesion was resected according to tumor-free technique rules—dissociating the tumor 2–3 cm apart from the reaction zone and truncating the bone 5 cm from the lesion. (2) The soft tissue and extraosseous tumor were cleared off, with the essential ligaments, like the collateral and lateral ligaments of the knee, retained. (3) The bone lesion was removed by bistrique and the remaining bone was immerged into 99 % alcohol for 30 min, then retrieved and flushed with 3000 ml physiological saline. (4) Kirschner combining with bone cement was used to fill the bone defects, and the final fixation was performed using the steel plate or intramedullary nail. Postoperative plaster immobilization was applied for 2 months and then removed. Patients were encouraged to do functional training with initial protection of the brace.

### Statistical analysis

Limb function was evaluated with the Musculoskeletal Tumor Society (MSTS) rating scales, which comprise six items, namely, pain, function, emotional acceptance, support, walking, and gait. Five points are allocated to each item and the highest score is 30 (100 %) [16]. Autografts that were functional and conserved were deemed as “survived,” and those that had been removed or had resorbed and were no longer functional were recorded “died.” Survival of patients was recorded using the Kaplan-Meier method with 95 % confidence interval.

## Results

**Fig. 1:**
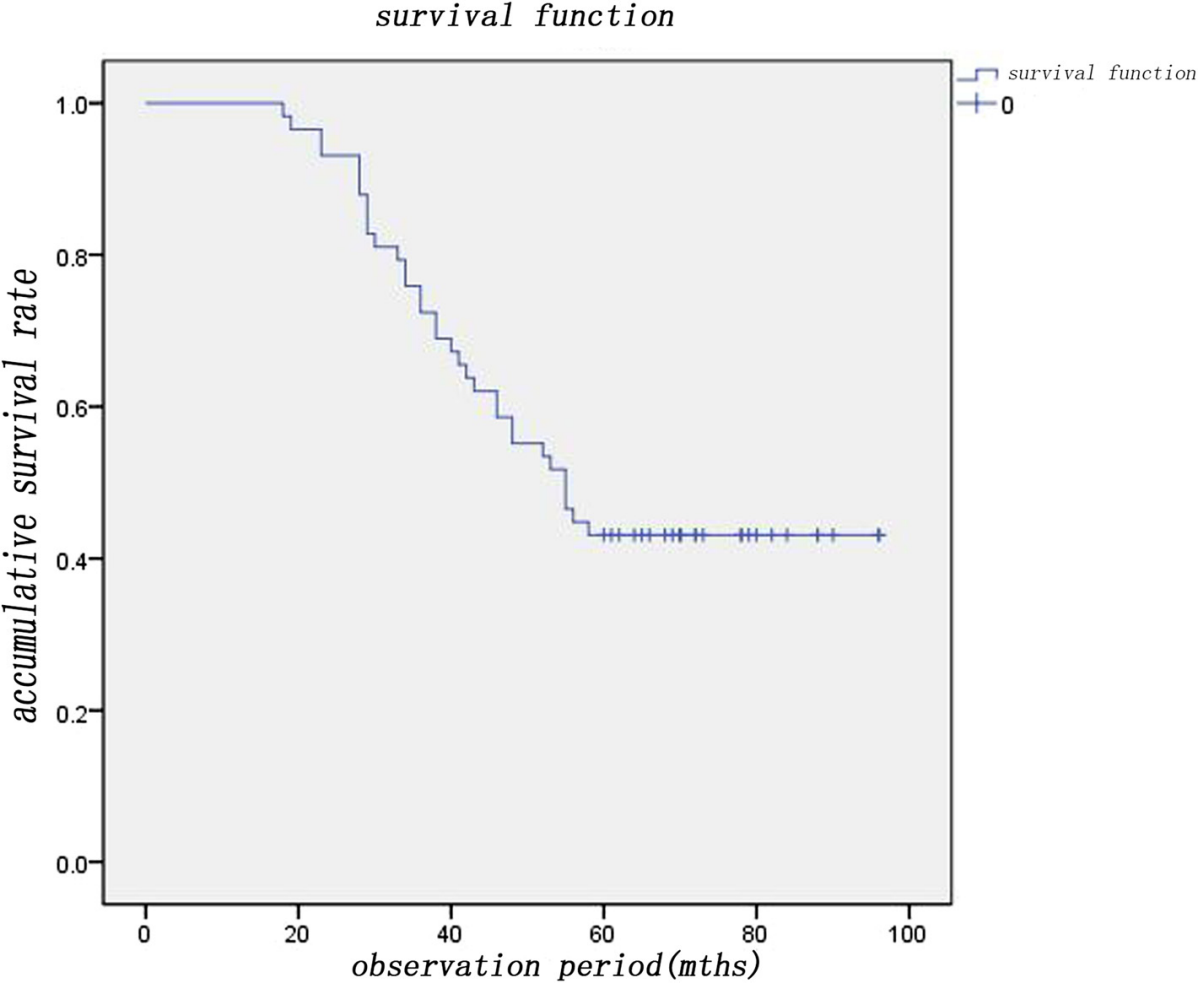
The 5-year survival rate. The mean survival period was 75.2 months (60–90), and 25 patients were alive and tumor-free, of which 16 osteosarcoma, 6 chondrosarcoma, 2 giant cell tumor of bone, and 1 mucus chondrosarcoma. Thus, the 5-year survival rate was 43.1 %. Sixteen patients died of lung metastasis, of which nine patients had local recurrence and lung metastasis (two patients received postoperative amputation and resection of lung metastatic foci). Eleven patients died of complications including infection, cachexia, and renal failure. Four patients died of other diseases and two patients died of reaction of chemotherapy

The mean survival period was 75.2 months (60–90), and 25 patients were alive and tumor-free, of which 16 osteosarcoma, 6 chondrosarcoma, 2 giant cell tumor of bone, and 1 mucus chondrosarcoma. Thus, the 5-year survival rate was 43.1 % (Fig. 1). Sixteen patients died of lung metastasis, of which 9 patients had local recurrence and lung metastasis (two patients received postoperative amputation and resection of lung metastatic foci). Eleven patients died of complications including infection, cachexia, and renal failure. Four patients died of other diseases and two patients died of adverse reaction of chemotherapy (Table 3). The mean MSTSS score was 78.5 % ranging from 47 to 98 %. Forty-nine autografts survived and nine died for several reasons including infection, necrosis, and absorption. In 58 patients, 3 were with local recurrence (5.2 %), 7 with lung metastasis (12.1 %), and 9 with both local and lung metastases (15.5 %).

**Table 3:** Complications of inactivated autograft treated with alcohol

Types (of complication)	No. (rates)
Early complications	11 (18.9 %)
Infection	8
Flap necrosis	3
Late complications	5 (8.6 %)
Fracture and union	3
Dislocation	2
Total	13 (22.4. %)

Complications were encountered in 13 of 58 patients (22.4 %), including deep infection in 8 (13.8 %), flap necrosis in 3 (5.2 %), fracture and nonunion in 3 (5.2 %), and joint dislocation in 2 (3.4 %) (Table 4). Eight patients with deep infection were managed by debridement, drainage, irrigation, and the use of antibiotics. The inactivated autograft was removed in two patients with deep infection. All the three local flap necrosis occurred in the proximal tibia, of which two cases healed after debridement and drainage and one case was with the infection out of control due to the patient automatically discharging from hospital. One patient with fracture of the autograft (due to trauma) was treated with secondary internal fixation. Two patients with fracture of steel plate received conservative treatment. Two patients with joint dislocation received prompt treatment. One was managed by resetting the joint and then with the plaster immobilization. The other was treated with removing the autograft and then filling the defects with bone cement instead.

**Table 4:** Outcomes of patients treated with inactivated autograft induced by alcohol

Types	No. (rates)
Alive or free of disease (≥5 years)	25
Death	33
Lung metastasis	16
Infection	4
Cachexia	4
Renal failure	3
Reactions of chemotherapy	2
Other diseases	4

## Discussion

Most bone tumor patients are young; thus, the treatments are supposed to not only preserve the limb but also maintain function without major complications or recurrences over long term [17, 18]. From a developing nation’s perspective, reimplantation of extracorporeally devitalized tumor-bearing bone segments is an appealing option. It allows immediate and anatomical correct filling of the defect [19]. Means of devitalizing tumor-bearing bone varies, including autoclaving, freezing, pasteurization, and extracorporeal irradiation. All the methods have similar effect in killing the tumor cells. However, the main differences lie in their effect on mechanical properties of the bone [20].

Since the first report of inactivated autograft (using alcohol) in the treatment of primary malignant musculoskeletal tumor by Song X. W. in 1983 [21], the method has been widely applied in hospitals throughout China. It has various advantages including low cost, no rejection or transmission of disease, no requirement for a bone bank or for special equipment, good fit between graft and host bone, and easy attachment of tendons and ligaments to the bone. In fact, it met the expectation of both the patients and the doctors, and some patients achieved decent long-term limb function [15, 22, 23]. However, this method was not well applied due to no uniform standard of selecting patients. Some patients that did not meet the criteria of limb salvage surgery were proposed to take this procedure, resulting in the failure of limb salvage, thus increasing the incidence of complications of the surgery objectively.

The bone shell inactivated by alcohol was almost dead. When it was replanted back to the host combining with bone cement, it takes more time to attach to the normal soft tissue and bone compared with the fresh one [24]. In the sites containing little soft tissue, like the proximal tibia, it is inevitably to be infected with the flap necrosis after the resection of tumor. In 11 patients with infection or flap necrosis, 9 occurred in the proximal tibia. There were several tips in coping with this situation. First, it is necessary to retain enough soft tissue; if not, the flap transferring surgery should been performed (the medial head of the gastrocnemius is most frequently used). Second, preoperative prophylactic use of antibiotics is essential and should be continued for a period time. Third, it is advisable to minimize the use of electric knife in resecting lesions next to the normal flap and avoid excessive traction of the flap. Fourth, adequate postoperative drainage is of great importance; thus, it is preferable to place unilateral or bilateral subcutaneous drainage strips and cover the gauze with pressure.

Though the tumor-bearing bone is autologous, it is indeed “dead” after the inactivation of alcohol. The healing process is similar to that of allograft, mainly through creeping substitution of host bone and infiltration of mesenchymal cells of soft tissue [25]. Therefore, this is a long-period process that some researchers believe to be 3 to 5 years [26]. One patient in our group experienced fracture due to trauma 6 years after the surgery. Intriguingly, when taking biopsy of the intraoperative cortical bone in the fracture site, it turned out to be without bone formation. However, the tumor-bearing bone still contained some active inducible factors for the limited penetration of alcohol, so the inactivated autograft takes less time to union compared with allograft [24]. In former animal experiments on the biomechanics and healing process of alcohol-inactivated bone, we found that the healing process initiated in the bone ends, followed by the middle section (which had the weakest mechanical strength in the late phase of healing) [26]. Therefore, to avoid fractures, the patients should not bear too much weight before complete clinical healing. For patients with fracture, secondary surgery of internal fixation was proposed if conditions are permitting. Otherwise, the conservative treatment was the wise choice. Different techniques have been proposed to reduce complications and improve functions of the affected extremities [27–29].

It was of great importance to enact replantation indications [15, 17, 30]. In our group, 12 cases were with local recurrence, of which 8 cases were the result of inappropriate selection of patients. Two patients with huge soft tissue mass showing poor response to chemotherapy were requested by their families to conduct limb salvage surgery, resulting in the failure of extensive resection of the tumor and subsequent recurrence 6 months later. The flap necrosis and infection occurred in a patient (with tumor in the proximal tibia) for retaining little soft tissue to cover the bone after surgery. Hence, selection criteria can affect the prognosis of patients and efficacy of limb salvage surgery directly. Our inclusion criteria of limb salvage surgery include the following: (I) tumors were sensitive to chemotherapy; (II) a limited boundary of the tumor; (III) good conditions for local soft tissue; (IV) a relative intact continuity of the resected bone; and (V) a good general condition, with no occurrence of other serious diseases. Patients with large tumors, unclear boundary, extensive invasion of soft tissue, or involvement of major blood vessels and nerves or who are insensitive to chemotherapy, cannot afford chemotherapy costs, or are reluctant to finish chemotherapy should be excluded.

Joint dislocation occurred mainly in the knee, which related to poor- or non-healing of ligament reconstruction [31]. The residual ligaments on inactivated autograft were “dead”, and the initial connection with normal ones was strengthened by sutures. The full healing of ligament could maintain a certain tension, preventing excessive sliding of the joint. If the reconstructed ligaments were absorbed or not healed, then the joint dislocation might occur. For patients with knee reconstruction and increased activity should be taken under the protection of brace. As for dislocation, closed reduction was the best choice. If the joints are dislocated for a long time, with the soft tissue contracted, the open reduction was proposed. For patients having difficulties in resetting the joint, the temporary support was essential until the secondary surgery with allograft or prosthesis.

Whether it was a semi-autogenous-inactivated joint replantation or a semi-allogeneic one, articular cartilage degeneration inevitably happened [32, 33]. The degree of degeneration was positively related to growing age, high frequency of exercise, long timespan after surgery, etc. In our group, patients receiving amputation in the secondary surgery showed serious degeneration of articular cartilage in postoperative anatomical specimens. Therefore, there is no effective method in alleviating the progression and degree of degeneration.

**Fig. 2:**
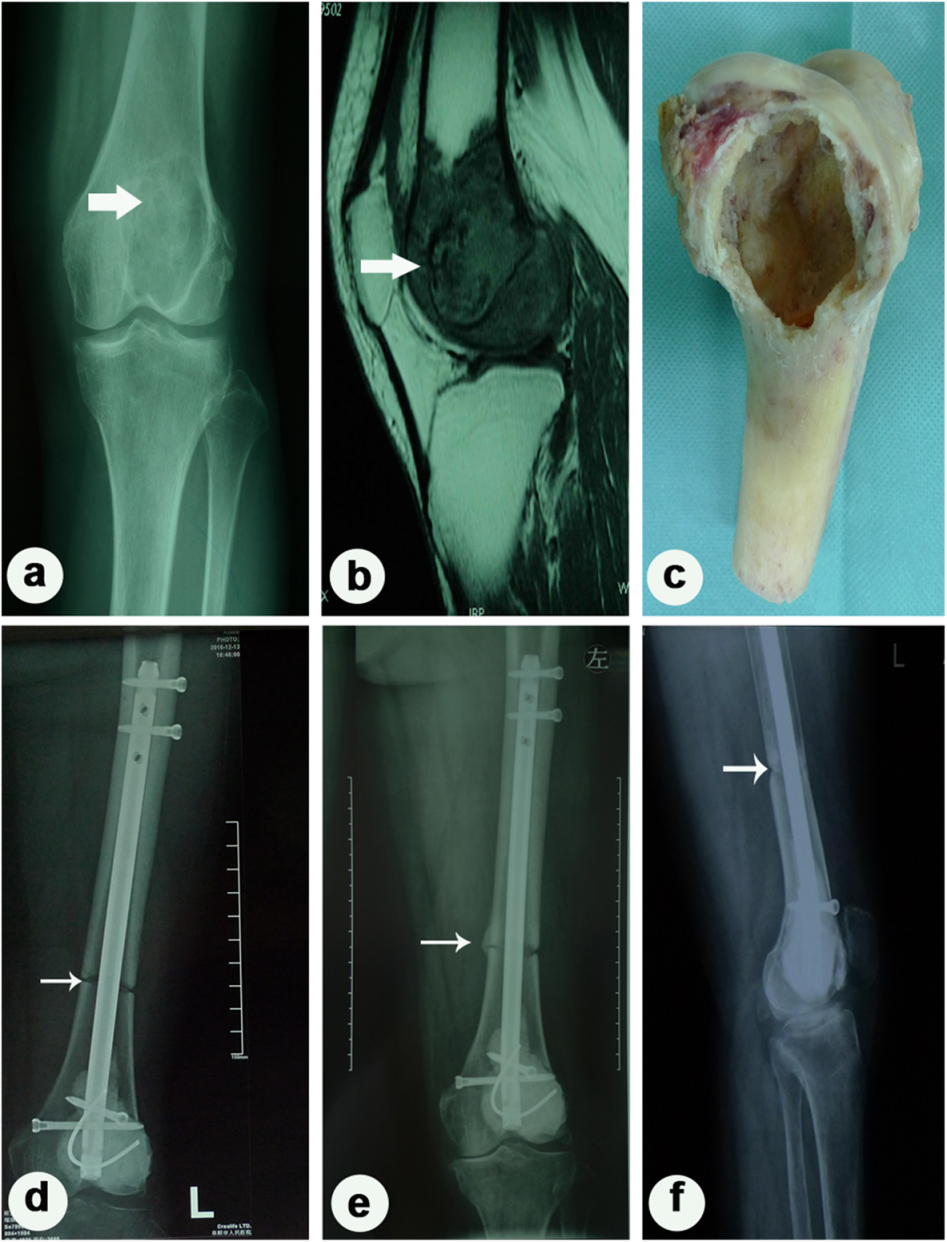
Case presentation I. Case 43: A 19-year-old man was diagnosed as having osteosarcoma in the distal of his left femur and was treated with wide resection and inactivated autograft using alcohol. **a** Plain radiography before surgery. **b** MRI before surgery. **c** Inactivated autograft by alcohol in surgery. **d** Three months after surgery. **e** One year after surgery. **f** Two years after surgery

**Fig. 3:**
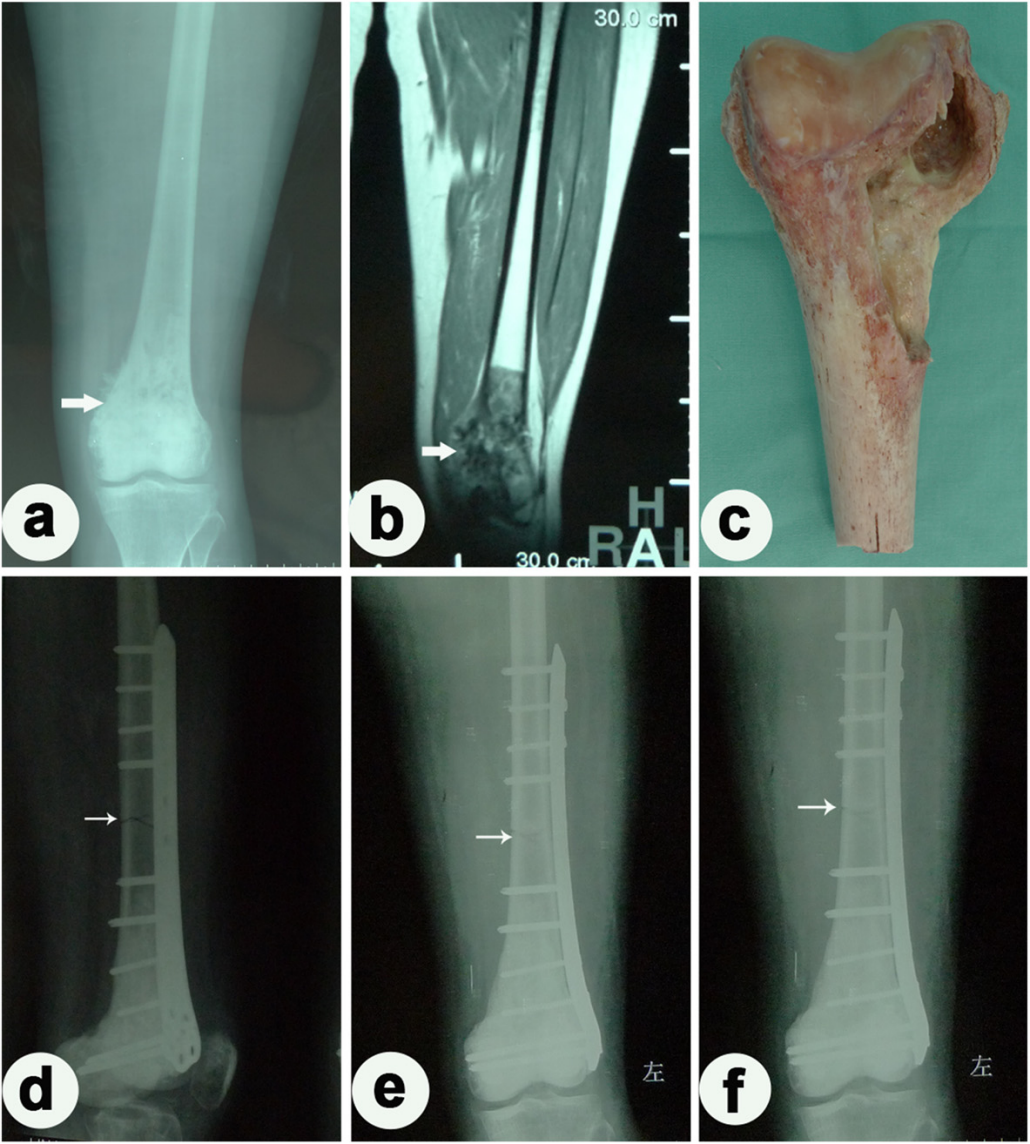
Case presentation II. Case 37: A 45-year-old woman was diagnosed as having chondrosarcoma in the distal of her left femur and was treated with wide resection and inactivated autograft using alcohol. **a** Plain radiography before surgery. **b** MRI before surgery. **c** Inactivated autograft by alcohol in surgery. **d** Three months after surgery. **e** One year after surgery. **f** Two years after surgery

The long-term outcome of our patients was poor due to multiple reasons, including the subjective and objective ones. For objective ones, it was inevitable. However, it was possible to eliminate the subjective ones. The latter patients of this group were screened strictly in accordance with the selecting criteria, and only four cases were with local recurrence. For patients who survived for a long term, the biological reconstruction using alcohol-inactivated autograft was an economic and effective alternative. In our group, some patients got a good outcome with a full heeling of autograft (Figs. 2 and 3). Anyway, all methods have their unfavorable aspects. Considering the relative high complication rates of this method, patients with indication of limb salvage can choose endoprosthetic treatment if it was economically affordable. For patients with lung metastasis, intensive involvement of vital vessels and nerves, or poor response to chemotherapy, amputation should be performed without hesitation.

## Conclusions

We find in this study that recycling autograft reconstruction using alcohol had favorable clinical outcomes to some extent. However, the rates of complications increased due to inappropriate selection of patients in the early period. After strictly adhering to indications of limb salvage, the rates decreased drastically. Therefore, the method should be used with caution in several aspects, especially in the indication of limb salvage surgery.
